# Traditional Herbal Medicine, Rikkunshito, Induces HSP60 and Enhances Cytoprotection of Small Intestinal Mucosal Cells as a Nontoxic Chaperone Inducer

**DOI:** 10.1155/2012/278958

**Published:** 2011-07-21

**Authors:** Kumiko Tamaki, Michiro Otaka, Tomoyoshi Shibuya, Naoto Sakamoto, Soh Yamamoto, Masaru Odashima, Hideaki Itoh, Sumio Watanabe

**Affiliations:** ^1^Department of Gastroenterology, Juntendo University School of Medicine, 2-1-1 Hongo, Bunkyo-Ku, Tokyo 113-8421, Japan; ^2^Department of Life Science, Faculty of Engineering and Resource Science, Akita University, Akita 010-8502, Japan; ^3^Department of Gastroenterology, Yokote Municipal Hospital, Akita 013-8602, Japan

## Abstract

Increasing incidence of small intestinal ulcers associated with nonsteroidal anti-inflammatory drugs (NSAIDs) has become a topic with recent advances of endoscopic technology. However, the pathogenesis and therapy are not fully understood. The aim of this study is to examine the effect of Rikkunshito (TJ-43), a traditional herbal medicine, on expression of HSP60 and cytoprotective ability in small intestinal cell line (IEC-6). Effect of TJ-43 on HSP60 expression in IEC-6 cells was evaluated by immunoblot analysis. The effect of TJ-43 on cytoprotective abilities of IEC-6 cells against hydrogen peroxide or indomethacin was studied by MTT assay, LDH-release assay, caspase-8 activity, and TUNEL. HSP60 was significantly induced by TJ-43. Cell necrosis and apoptosis were significantly suppressed in IEC-6 cells pretreated by TJ-43 with overexpression of HSP60. Our results suggested that HSP60 induced by TJ-43 might play an important role in protecting small intestinal epithelial cells from apoptosis and necrosis in vitro.

## 1. Introduction

Recently, it has been recognized that small intestinal ulcers and bleeding are not rare complications of nonsteroidal anti-inflammatory drugs (NSAIDs), since capsule endoscopy and balloon enteroscopy are available for the detection of small intestinal lesions. Graham et al. have reported that 71% of rheumatoid arthritis patients receiving NSAIDs for at least 3 months had small intestinal mucosal lesion [1]. Sugimori et al. also have reported that more than 50% of patients taking NSAIDs have some mucosal damage in the small intestine [2]. Sometimes, intestinal bleeding could be a lethal complication [3]. Population who will take NSAIDs for cerebrovascular diseases and ischemic heart diseases is easily predicted to increase in aging society in future. Therefore, development of their treatment and prevention are seriously required in clinical field. However, much about neither the pathogenesis nor therapy is well understood compared with that in the gastric mucosal lesions associated with NSAIDs.

Many studies have shown the importance of heat shock proteins (HSPs, molecular chaperones) for survival of cells under stress conditions [4, 5]. It has been focused on the cytoprotective functions of HSPs, which include folding or assembly of degenerated proteins to normalize intracellular environment, under stressed conditions mediated by chaperon function of HSPs. These functions are considered to be essential for living cells to obtain tolerance to adapt to environmental changes [6, 7]. Cytoprotective functions of a 70-kDa-heat shock protein (HSP70) and a 60-kDa-heat shock protein (HSP60) have been demonstrated in several experimental models including the stomach and colon [8–13]. In the gastric mucosa, some antiulcer drugs such as geranylgeranylacetone (GGA) or zinc L-carnosine have been reported to induce gastric mucosal HSP70 and enhance mucosal protective ability both in vitro and in vivo [11, 14]. These “nontoxic chaperone inducers” are considered to be new therapeutic strategy for gastric mucosal injury. We previously have reported that when HSP60 was overexpressed by cDNA transfection in the intestinal epithelial cells, the cytoprotective ability could be dramatically enhanced [15].

Rikkunshito (TJ-43) is a traditional Japanese herbal medicine used for dyspeptic symptom and chronic gastritis. In the present study, we aimed to evaluate whether TJ-43 could have therapeutic value for therapy of NSAID-induced mucosal damage as a nontoxic chaperone (HSP60) inducer in the small intestinal cells.

## 2. Subjects and Methods

### 2.1. Rat Small Intestinal Cell Line

A rat small intestinal epithelial cell line (IEC-6) was obtained from Riken Cell Bank (Tsukuba, Japan). This cell line was cultured in 1640 RPMI medium (Gibco, NY, USA) with 10% fetal bovine serum at 37°C in a CO_2_ incubator.

### 2.2. Effect of TJ-43 on HSP60 Expression in IEC-6 Cells

IEC-6 cells were treated with 2.0 mg/mL of TJ-43 in serum-free medium for 2 h. This condition is effectively induces HSP60 without cytotoxicity in IEC-6 cells. IEC-6 cells were harvested 6 h after TJ-43 exposure. The cells were homogenized with 5 volumes of ice-cold 25 mM Tris-Cl buffer (pH 7.5). The homogenates were centrifuged at 15,000 ×g for 10 min. The protein concentration was measured by the method of Bradford [16]. Samples were analyzed by 9% polyacrylamide gel electrophoresis according to the method of Laemmli [17]. Briefly, samples (20 *μ*g/lane) were electrophoresed on 9% SDS-polyacrylamide gels, transferred electrophoretically to PVDF membrane (removal rating, 0.45 *μ*m; Amersham Pharmacia Biotech, Buckinghamshire, England), and processed as described by Towbin et al. [18]. The membrane was incubated with anti-HSP60 antibody (StressGen, Victoria BC, Canada) and treated with horseradish peroxidase conjugated antirabbit IgG (1 : 1500) (Bio-Rad, Richmond, Calif., USA). As an internal control, glyceraldehyde-3-phosphate dehydrogenase (GAPDH) was detected by anti-GAPDH antibody (Santa Cruz, Calif., USA). The stained bands were visualized with 0.02% 3, 3-diaminobenzidine tetrahydrochloride containing 0.005% hydrogen peroxide. The density of the immunologically stained band was analyzed by scanning densitometer using Multi-Gauge (ver 3.0) (Fujifilm, Tokyo, Japan) program according to the method we previously reported [13]. The relative density of HSP60 was calculated using next formula: relative density (%) = density ratio (HSP60/GAPDH, after TJ-43 treatment)/density ratio (HSP60/GAPDH, before TJ-43 treatment)×100.

### 2.3. Effect of TJ-43 on Antinecrotic Ability of IEC-6 Cells

IEC-6 cells were seeded into 96-well flat-bottom culture plates at a density of 3×10^4^/mL and incubated for 24 h after exchanging the medium to serum-free medium. Cytoprotective ability of IEC-6 cells against hydrogen peroxide- (0–0.18 mM) or indomethacin- (0–2.4 mM) induced cell damage was analyzed with or without pretreatment with 2.0 mg/mL of TJ-43 for 2 h [12, 15]. The cell viability was measured by modified MTT (3-(4,5-di-methylthiazol-2-yl)-2,5-diphenyltetrazolium bromide, yellow tetrazole) assay (CellTiter 96 Nonradioactive Cell Proliferation Assay, Promega, Wis, USA) 6 h after exposure of the cells to hydrogen peroxide or indomethacin. The cell survival (%) was evaluated using next formula. Relative cell survival (%) = [absorbance (in each hydrogen peroxide or indomethacin concentration)/absorbance (at 0 mM of hydrogen peroxide or indomethacin)] ×100. Cytoprotective ability of IEC-6 cells against hydrogen peroxide- (0–0.4 mM) or indomethacin- (0–4.0 mM) induced direct plasmamembrane damage was analyzed by lactate dehydrogenase (LDH)-release assay using a CytoTox 96 Nonradioactive Cytotoxicity Assay (Promega, Wis, USA), with or without pretreatment with TJ-43. The relative (%) cell necrosis was evaluated using the next formula. Relative cell necrosis (%) = [absorbance (in each hydrogen peroxide or indomethacin concentration)/absorbance (maximum LDH-release)] ×100.

### 2.4. Effect of TJ-43 on Antiapoptotic Ability of IEC-6 Cells

Antiapoptotic abilities of IEC-6 cells and IEC-6 cells treated with TJ-43 were compared based on MTT assay, TUNEL staining, and caspase-8 activity after exposure of these cells to low concentration of hydrogen peroxide (0–0.16 mM) or indomethacin (0–1.0 mM) for 18 h in serum-free medium [12, 15]. The relative cell survival (%) was evaluated by MTT assay and the next formula. Relative cell survival (%) = [absorbance (in each hydrogen peroxide or indomethacin concentration)/absorbance (at 0 mM of hydrogen peroxide or indomethacin)]×100. Apoptotic cells were fluorescence microscopically detected by TUNEL (tdT-mediated dUTP nick-end labeling) staining (DeadEnd Fluorometric TUNEL System, Promega, Wis, USA) in green color fluorescence in a blue background of all cells. The ratio of apoptotic cells was counted by two observers who were blind to the group.

The caspase-8 activity in the cells was measured using Caspase-Glo 8 Assay kit (Promega, Wis, USA) after exposure of IEC-6 cells to hydrogen peroxide (0–0.1 mM) or indomethacin (0–0.6 mM) with or without pretreatment with TJ-43.

### 2.5. Data Analysis

All data were expressed as means ± SEM with at least three separate experiments using more than six samples in each experiments (*n* > 18). Statistical analysis was performed using Student's *t*-test, where it was appropriate, after ANOVA analysis; *P* < 0.05 was considered to be statistically significant.

## 3. Results

### 3.1. Expression of HSP60 after TJ-43 Treatment

**Figure 1:**
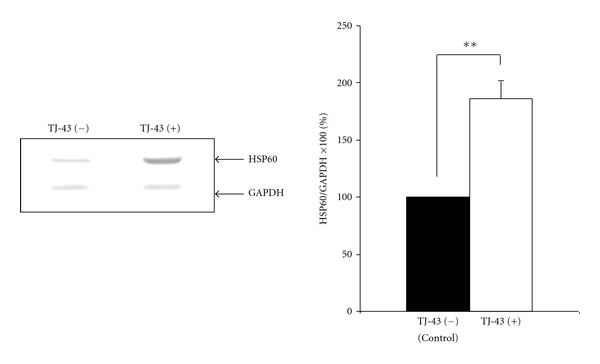
Western blot analysis. TJ-43 significantly induced the expression levels of HSP60 in IEC-6 cells **P < 0.01 compared with control cells.

As shown in Figure 1, TJ-43 significantly induced HSP60 expression (approximately 2-fold) compared with that in IEC-6 cells determined by immunoblotting with quantification by densitometric data normalized by internal control protein (GAPDH) (*P* < 0.01).

### 3.2. Effect of TJ-43 on Cytoprotective Ability of IEC-6 Cells

#### 3.2.1. Antinecrotic Ability

**Figure 2:**
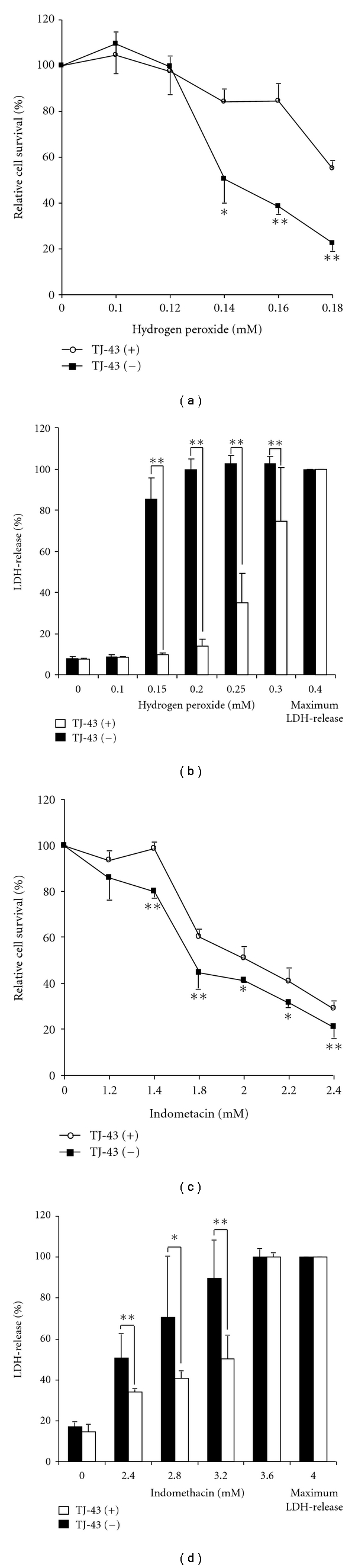
Antinecrotic ability. Hydrogen peroxide-(a) or Indomethacin-(c) induced cell necrosis was significantly suppressed in IEC-6 cells pretreated with TJ-43 compared with nontreated IEC-6 cells under necrotic condition based on MTT assay. Cytotoxicity, based on LDH-release after hydrogen peroxide- (b) or indomethacin- (d) treatment was also significantly reduced in TJ-43 pretreated IEC-6 cells compared with nontreated IEC-6 cells (control cells). Values are means ± SEM. *P < 0.05, **P < 0.01 compared with control cells.

As shown in Figure 2(a), hydrogen peroxide-induced cell necrosis was significantly suppressed in IEC-6 cells pretreated with TJ-43 compared with nontreated IEC-6 cells under necrotic condition based on MTT assay. IEC-6 cells treated with TJ-43 also showed greater viability against indomethacin-induced cell necrosis compared with nontreated IEC-6 cells (Figure 2(c)). The direct plasma membrane damage (necrosis) based on LDH-release was also significantly reduced in TJ-43 pretreated IEC-6 cells compared with nontreated IEC-6 cells (Figures 2(b) and 2(d)).

#### 3.2.2. Antiapoptotic Ability

**Figure 3:**
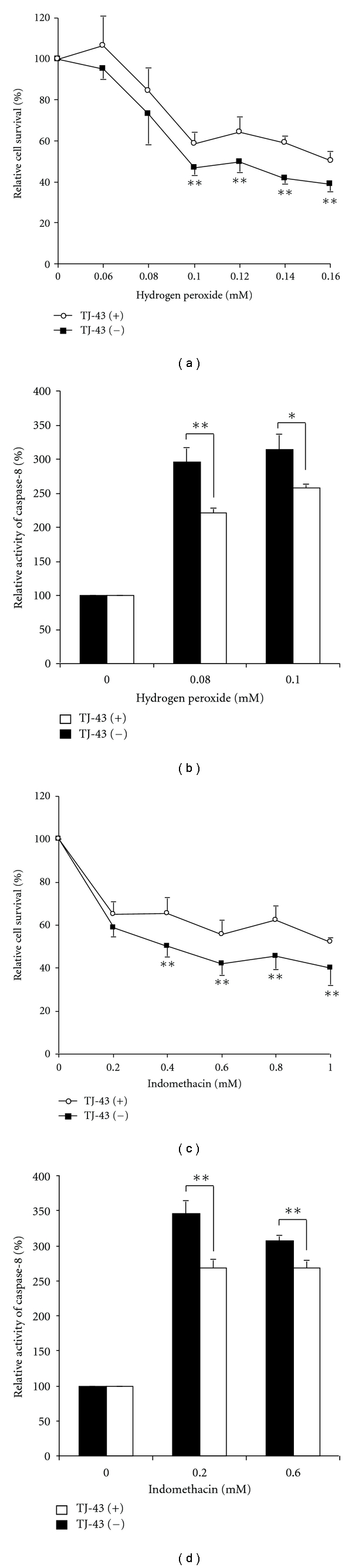
Antiapoptotic ability. Hydrogen peroxide- or indomethacin-induced apoptosis evaluated by MTT assay (a, c) and caspase-8 activity (b, d) was significantly suppressed in TJ-43 pretreated IEC-6 cells compared with nontreated IEC-6 cells (control cells). Values are means ± SEM. *P < 0.05, **P < 0.01 compared with control cells.

**Figure 4:**
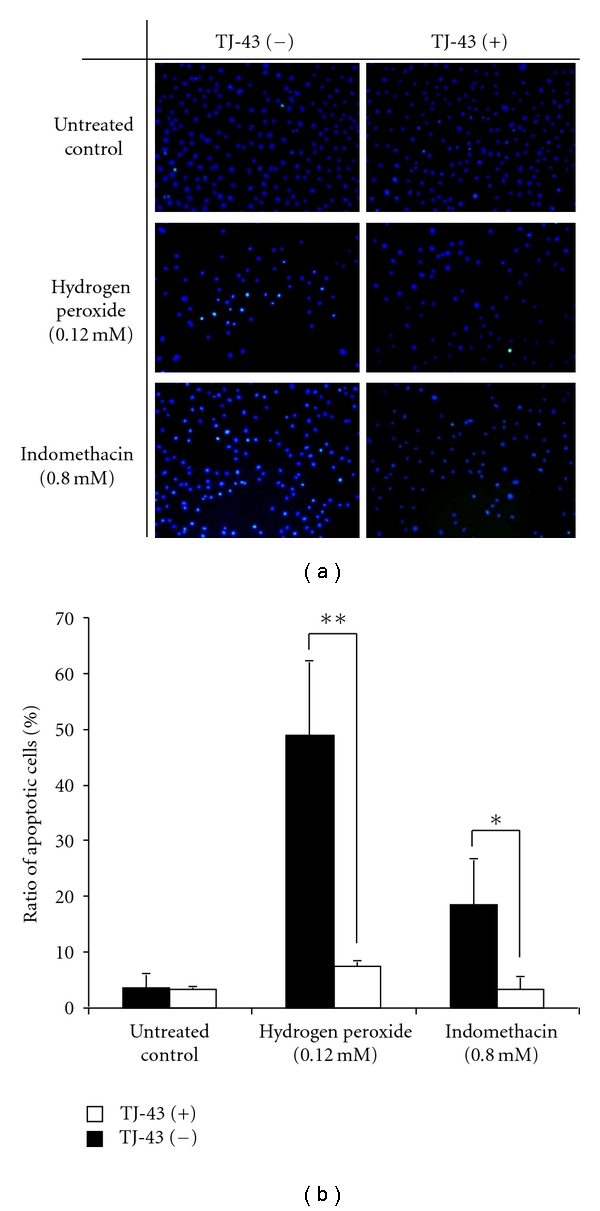
TUNEL staining. (a); IEC-6 cells treated with hydrogen peroxide or indomethacin showed typical morphological features of apoptotic cells with condensed and fragment nuclei. When IEC-6 cells were pretreated with TJ-43, apoptosis was apparently suppressed. (b); Ratio of apoptotic cells significantly reduced in IEC-6 cells treated with TJ-43 compared with control cells (untreated IEC-6 cells). Values are means ± SEM. *P < 0.05, **P < 0.01 compared with control cells (IEC-6-mock cells).

As shown in Figures 3(a) and 3(c), hydrogen peroxide- or indomethacin-induced apoptosis was significantly suppressed in IEC-6 cells pretreated with TJ-43 compared with nontreated IEC-6 cells based on the cell survival. Activity of the caspase-8 was significantly reduced in IEC-6 cells treated with TJ-43 compared with nontreated IEC-6 cells (Figures 3(b) and 3(d)). To characterize the cell death induced by hydrogen peroxide or indomethacin, nuclear morphology of apoptotic cells was also examined with a fluorescent DNA-binding agent (TUNEL staining). Cells treated with hydrogen peroxide or indomethacin showed typical morphological features of apoptotic cells with condensed and fragmented nuclei (Figure 4(a)). Ratio of apoptotic cells was significantly reduced in IEC-6 cells treated with TJ-43 compared with untreated control cells (Figure 4(b)).

## 4. Discussion

Results of our present study clearly demonstrated the enhancement of cytoprotective ability in IEC-6 cells pretreated with TJ-43 to induce HSP60 (molecular chaperone) compared with nontreated control cells. The enhanced cytoprotective ability was observed under both necrotic and apoptotic conditions induced by hydrogen peroxide or indomethacin. 

NSAIDs are widely used in many clinical fields such as cardiovascular, rheumatic, and orthopedic diseases. Recent advances in diagnostic methods, including capsule endoscopy and double balloon enteroscopy, have enabled to examine the entire small intestine, and it is now acknowledged that small intestinal lesions are not rare as considered before [1, 2, 19, 20]. Gastrointestinal bleeding is one of the most serious well-known adverse reactions of NSAIDs. Gastric mucosal lesion is a well-investigated complication that is mainly caused by cyclooxygenase (COX)- 1 inhibition resulting in decrease of prostaglandin E2 in the gastric mucosa [21, 22]. Also, gastric acid has been reported to be an exacerbation factor of NSAID-induced gastric mucosal damage [23]. Therefore, prostaglandin derivatives and proton pump inhibitors (PPI) have been considered as evidence-based therapeutic tools for NSAID-induced gastric mucosal injury [24, 25]. Further, as internal gastric mucosal protectant, molecular chaperone, especially HSP70, has been reported to be a candidate of therapeutic tool for NSAID-induced gastric mucosal lesion [10]. In order to induce gastric mucosal HSP70, some nontoxic chaperone inducers have been developed such as GGA and zinc-L-carnosine which are commercially available antiulcer drugs used for gastritis and gastric ulcer in Japan and some Asian countries [11, 13, 14]. As an induction mechanism of HSP, we have reported that heat shock transcription factor 1 (HSF-1) was activated by these drugs [26]. These antiulcer drugs predominantly increase gastric mucosal protective ability against NSAIDs, ethanol, and hydrogen peroxide [8, 14]. On the other hand, little has been known about the pathogenesis and therapy of small intestinal lesions induced by NSAIDs. It has been reported that PPI or prostaglandin E may not have sufficient effect for small intestinal damage [27, 28]. Although HSP70 is an important molecular chaperone in the gastric mucosa as mentioned above, HSP60 has been reported to mainly contribute to small intestinal mucosal protection whereas induction of HSP70 did not enhance the protective ability [15]. Therefore, it is considered that medications that increase HSP60 in the small intestinal epithelial cells could be new therapeutic tools for small intestinal mucosal damage. 

TJ-43 is a traditional Japanese herbal medicine extracted from a mixture of Atractylodis Lanceae Rhizoma, Ginseng Radix, Pinelliae Tuber, Hoelen, Zizyphi Fructus, Aurantii Nobilis Pericarpium, Glycyrrhizae Radix, and Zingiberis Rhizoma [29]. TJ-43 has been reported to improve dyspeptic symptom by reversing an existing impaired adaptive relaxation [29–31], leading to an improvement of delayed gastric emptying mediated by ghrelin receptor and 5-HT_2_ receptor antagonism [29–32]. Also, some herbal medicines inducing TJ-43 have been reported to protect gastric mucosal epithelial cells from oxidative injury. One of the mechanisms for mucosal protection has been considered to be mediated by their antioxidant activities as a radical scavenger [33]. 

We previously have reported that when HSP60 is overexpressed by cDNA transfection technique, the cytoprotective ability is enhanced in the small intestinal mucosal cell [15]. However, in view of clinical application of chaperone-inducing therapy, cDNA transfection may not be practical. It could be convenient if nontoxic drugs could effectively induce HSP60 in the small intestinal mucosa. In the present study, TJ-43 exerts protective effects of the small intestinal epithelial cells against not only oxidative injury but also NSAID-induced cell damage with increase in the level of HSP60. These results indicate that TJ-43 can be a “nontoxic chaperone (HSP60) inducer” in the small intestine.

## 5. Conclusion

The present paper provides the first evidence of mucosal protective effect of TJ-43 from small intestinal damage induced by indomethacin or oxidative injury with induction of molecular chaperone (HSP60). In the therapeutic point of view, it is suggested here that “chaperone-inducing herbal medicine therapy” could be an important candidate of a new strategy for small intestinal mucosal injury including inflammatory bowel diseases and NSAID-induced small intestinal mucosal injury.
